# “Nick Nax”

**Published:** 1859-06

**Authors:** 


### "NICK NAX,"

By generating merriment, banishing "blues," and provoking laughter weekly in New York, at ten cents hebdominally, one dollar a year, is not a theoretical, but a practical hygenian, the most efficient health promoter in our present memory, our own pet Journal excepted. We beg however, with all due respect to inquire if it is poking fun at us, our wisdom, or our practice by the following perpetration in a recent number? Meanwhile we will pause for a reply.

How to Doctor a Prima Donna.—Dr. Hall of this city, is the subject of a good story. Dr. H. is one of those gentlemen who can see a long way into a millstone. He is a gentleman whose reputation is established, and does only what is called an "office practice." By this is meant that his patients came to him instead of him going to them. Some days ago—we don't care to be more specific—a certain opera company was performing in this city. The "Bohemian Girl" was advertised, when the manager received a note from the prima donna, stating that she was sick, and could not possibly appear. This announcement came down like a cold water douche in January. The manager exclaimed;

"Don't believe it! An infernal lie fabricated to annoy me."

Manager called upon prima donna, and found her lying on a sofa. Manager forced an amiable look, and suggested a doctor. Prima donna allowed she had no objection. Manager rushed out and sent for Dr. H. He came about an hour afterwards, and found prima donna alone. Dr. H. felt prima donna's pulse, looked at prima donna's tongue, and allowed that prima donna was a foo-foo. Dr. H. ordered up an inkstand and wrote something on a slip of paper. Dr. H. handed paper to prima donna, and requested her to give it to manager. Prima donna spoke very little English, but with the assistance of her maid managed to comprehend the message. Just as Dr. H. had gone, manager came in rubbing his hands and smiling like a bunch of hollyhocks.

"Well, madam, what did Dr. H. say?"

Prima donna languidly pointed to the supposed prescription, which read:

"To deprive a prima donna of her health, neglect to pay her salary for a week. To restore it, settle up, and add flattery to atone for the neglect."

It remains only to say that Dr. H. hit the nail on the head. The manager *was* in arrears to prima donna. Manager saw how matters stood and rushed out. He returned in half an hour minus his watch and diamond ring, which he had "spouted" in order to raise the necessary funds. The result was exactly as Dr. H. had predicted. Having placed the money in a gold-gilt porte monnaie, prima donna rallied, and in ten minutes afterward was on manager's arm, going to rehearsal. It is rather a pity to add that manager's watch and ring are still in chancery.

